# Monitorization of Autonomic Stress Response of Nurse Students in Hospital Clinical Simulation

**DOI:** 10.1155/2021/6641425

**Published:** 2021-02-18

**Authors:** Ana Isabel Beltrán-Velasco, Paula Sánchez-Conde, Domingo Jesús Ramos-Campo, Vicente Javier Clemente-Suárez

**Affiliations:** ^1^Languages and Education Faculty, Antonio de Nebrija University, Madrid 28015, Spain; ^2^Faculty of Sport Sciences, Universidad Europea de Madrid, 28670 Villaviciosa de Odón, Spain; ^3^Sport Science Faculty, Catholic University of Murcia, Murcia 30107, Spain; ^4^Grupo de Investigación en Cultura, Educación y Sociedad, Universidad de la Costa, Barranquilla 080002, Colombia

## Abstract

The aims of this study were to analyze the autonomic stress response of nurse degree students during a hospital clinical simulation and to analyze differences in the autonomic stress response of nurse degree students during a hospital clinical simulation depending on their psychological profile. We analyzed in 45 nurse students their psychological profile (purpose in life, coping flexibility, perceived stress, Framingham Type A Behavior, and personality) and the autonomic modulation by the heart rate variability in a hospital clinical simulation. Students presented decreased heart rate variability and different autonomic stress responses depending on the different psychological parameters evaluated. We concluded that a hospital clinical simulation produced a large sympathetic modulation of nurse students that was maintained during the entire clinical simulation. The autonomic response was modulated by the psychological profile of students, showing higher purpose in life, perceived stress, and neuroticism, presenting higher parasympathetic modulation.

## 1. Introduction

The implementation of hospital clinical simulations has been marked as essential in the Health Science higher education learning process [1]. The evidence of the negative effects of the stress response in uncontrolled environments has been studied in other fields showing how the repeated exposure to these contexts facilitates the process of habituation in students [2] and, therefore, the improvement of the teaching-learning process [3, 4]. On the contrary, other studies found how in some stressful academic contexts, the expected habituation was not reached, maintaining students a large stress response, being a focus of uncontrollability that could affect academic performance [5]. In this line, the nurse degree students are subjected to repeated learning activities and evaluations in hospital clinical simulation environments [6–10]. These scenarios are designed by practitioners and professors to provide students the necessary skills for their professional practice, integrating theoretical and practical knowledge. In these simulation contexts, students must be able to apply previous theoretical knowledge in a stressful environment. In the area of Health Science studies, the simulation provides to the student a controlled clinical environment that is as real as possible in order to appear in a clinical context. This academic context provides students skills related to data collection, analysis, and monitoring of the patient's basic parameters. Simulations not only facilitate learning in the academic environment but also improve students' confidence and complex decision-making. Student behavior is influenced by the teaching-learning process that facilitates this controlled clinical experience, being modulated by the student behaviors in the professional setting [11].

The exposition to these uncontrolled and unpredictable scenarios could produce different effects in the participants' psychophysiological response, increasing the sympathetic nervous system modulation, negatively affecting memory and perceived exertion [12, 13]. The way to deal with stressful situations is also determined by the personal psychological profile. The nursing students must face situations of high emotional intensity, crisis situations, physical and emotional exhaustion, and feelings such as anger, frustration, or despair of patients attended [14]. In this line, it is important to know the psychological profile of the students to learn how they confront these situations during the training process and in their professional practices [15]. Certain personalities would define the coping style with contextual stressors and the tools used in these unpredictable contexts [16]. Psychological characteristics of cognitive flexibility, resilience, or collaborative work are associated with worse stress management, being able to have a negative impact on students' academic performance, interfering with the necessary skills needed during clinical simulation [17].

The objectives of the present study were as follows: (i) to analyze the autonomic stress response of nurse degree students during a hospital clinical simulation and (ii) to analyze differences in the autonomic stress response of nurse degree students during a hospital clinical simulation depending on their psychological profile. The initial hypotheses were as follows: (i) students would show a habituation process, decreasing the sympathetic nervous system activity at the end of the hospital clinical simulation; and (ii) the autonomic stress response would be modulated by the psychological profile of students.

## 2. Material and Methods

In order to reach the study objectives, an intentional opinatic sampling was carried out in a second-year nurse degree student group.

### 2.1. Participants

We studied 45 students of the nursing degree: 28.8% of men and 71.1% of women. They were between 19 and 40 years old (*M* = 26.48; SD = 6.86). The sample size calculation for the present research with a 14% margin of error and 95% confidence level was 40 subjects. The inclusion criteria were that participants were nurse degree students. Exclusion criteria were the presence of any medical condition or injury at the moment of the evaluation and the intake of any medicine, drug, dietary supplement, stimulants, or other ergogenic aids 24 h prior to the evaluation.

The whole procedure was done following the Declaration of Helsinki (revised in Brazil, 2013) and approved by the ethics committee of the university. The data were collected anonymously. Before starting the study, all participants were informed about the process to be carried out and informed of the right to withdraw from the research project at any time. To collect this signed informed consent, a live information session was organized with all students who could be part of the study. They were explained in detail the study objectives, that it was voluntary, and that they could withdraw at any time. In addition, the assessment methodology and instruments of the study were presented, and students were able to interact with them and test them. Once everything was explained, the students who wanted to participate signed the document and accepted to participate voluntarily. All students participating in the project stated that they had not faced a clinical simulation before. In addition, they had not been evaluated with this methodology either. Then, they presented the same experience in clinical sessions, so the acquisition of skills was done under the same conditions.

### 2.2. Design and Procedure

We conduct procedures used by the previous literature in the psychophysiological evaluation [1]. Firstly, we analyze the autonomous nervous system response during a clinical simulation scenario performed by nursing students. For this aim, we evaluated 6 parameters of heart rate variability during 5 specific moments along with the scenario: M0 (5 min presimulation moment); M1 (interval corresponding to the first tenth of the hospital clinical simulation); M2 (interval corresponding to the fifth tenth of the hospital clinical simulation); M3 (interval corresponding to the tenth tenth of the hospital clinical simulation); and M4(5 min postsimulation moment) (Table 1). The basal pre- and postsimulation moments were conducted in a separated room, free of noises and distractions, with controlled temperature and humidity. Participants were seated in a chair without talking and with the instruction to relax with calm respiration. The temperature and humidity of the hospital clinical simulation and the hospital room when pre- and postsamples were taken were22.1 ± 0.2°Cand44.3 ± 0.3%, respectively. All the simulations were conducted between 10:00 and 12:30 AM.

We also analyzed the subjective perception of stress before and after the hospital simulation using a scale of subjective distress units (SUDS); the students perceived the academic performance (grade on the simulation) and the teacher grade of the simulation; and the psychological variables include Life Engagement Test, Coping Flexibility Scale, Perceived Stress Scale, Framingham Type A Behavior Scale, and NEO-FFI.

The study started by placing in each student a heart rate monitor 5 minutes before the start of the exposure (M0). At this point, the student is alone in a quiet room with a controlled temperature (25.1 ± 0.2°C). Then, each student moves with a teacher who guides him/her to the clinical simulation scenario and receives a brief instruction. The scenarios were previously designed by a group of experienced teachers (associate professors of the subject) where they took into account each pathology presented, the materials available, the necessary skills, and the fundamental aspects to evaluate the student. With this information, an objective performance evaluation scale was developed to analyze every student with the same parameters. During the clinical simulation, the professor cannot help students and impartially evaluated the students' actions.

Once the students started the scenario, they had 15 minutes to deal with an unknown situation but previously studied during the academic year.

#### 2.2.1. Clinical Simulation

The scenario evaluated in the present research was a medical emergency where nurse students must interact with a simulated patient (actor) and other health professionals (actors).

In the simulation scenario, a 61-year-old male patient is admitted to the emergency department for an unknown pathology. He presents with abdominal pain extending to the back for the past week. In addition, he has had a fever for two days and has vomited occasionally the previous night. This is the third time he has visited the emergency department in the last week. He was diagnosed with a urinary tract infection and was prescribed antibiotics and a fever reducer. However, the pain persists, so he comes to the hospital again.

### 2.3. Study Variables and Instruments

*Autonomic modulation* was analyzed by the monitorization of heart rate variability (HRV). The R-R interval of the heartbeat was used as a measure of the autonomous modulation presented by the participants. This recording was made with a Polar V800 heart rate monitor (Polar, Kempele, Finland) following the indications of the previous literature [18]. The R-R series was analyzed with the Kubios HRV software (version 2.1, Biosignal Analysis and Medical Imaging Group, University of Kuopio, Finland), following the recommendations of the existing scientific literature [19]. This software has been verified, and its efficacy in recording time intervals between heartbeat (R-R) variations has been demonstrated to be extremely accurate [20]. A very low threshold of artifact correction was applied, obtaining 0.09% of artifact correction. The frequency bands used for the AR spectrum analysis were LF (0.04-0.15 Hz) and HF (0.15-0.4 Hz). The HRV parameters analyzed were the following: LF: the low-frequency band in standardized units (LF n.u.); HF: the high-frequency band in standardized units (HF n.u.); LF/HF ratio: the low-frequency/high-frequency ratio; PNN50: the number of successive differences of the intervals differing by more than 50 ms expressed as a percentage of the total; RMSSD: the square root of the mean sum of the squared differences between adjacent normal R-R intervals; SD1: sensitivity of the short-term variability of the nonlinear spectrum of the HRV; and SD2: sensitivity of the long-term variability of the nonlinear spectrum of the HRV.

#### 2.3.1. Subjective Perception of Distress is Trait

A scale of subjective stress perception units (SUDS) with scores from 0 to 100 was used. These units indicate a level of anxiety perceived by the subject at the time of the assessment, which goes from zero (0), which implies “completely indifferent and cold; it does not affect me,” to one hundred (100), which means “so distressed and tense that I cannot cope.” This scale will be applied to the participating subjects five minutes before the beginning of the exposure to the simulation and will provide information about the level of stress perceived by the individual at this moment and represents the cognitive relationship between the objective event and the emotional response [21].

#### 2.3.2. Life Engagement Test (LET)

This scale is composed of 6 items and was designed to measure the purpose in life. This scale analyzes the degree of commitment that a person has in the performance of different activities that can be defined as important within his/her daily environment. An example item is as follows: “For me, all the things I do deserve punishment.” This test is answered on a Likert scale from 1 to 5, where 1 = totally disagree and 5 = totally agree [22].

#### 2.3.3. Coping Flexibility Scale (CFS)

This scale was designed to measure a person's flexibility to cope with different situations and will provide information about the presence and use of adaptive strategies that are historically associated with better psychological health. This test has 10 items and is answered on a Likert scale from 1 to 4, with 1 = very applicable and 4 = not applicable. An example item is as follows: “I am aware of the success or failure of my attempts to cope with stress” [23].

#### 2.3.4. Perceived Stress Scale (PSS)

This scale evaluates the level of perceived stress over a one-month period. It is composed of 14 items that are answered on a five-point Likert scale, where 0 = never and 4 = very often. An example item is as follows: “In the last month, how many times have you felt that you had everything under control?” High scores are related to increased perception of stress [24].

#### 2.3.5. Framingham Type A Behavior

This questionnaire consists of 10 items which assess the personality type associated with certain behaviors that differ from conventional patterns, as well as its relationship with different health anomalies. The questions were responded in a Likert-type scale of five points, where 1 = never and 5 = very often [25].

#### 2.3.6. NEO-FFI

This scale assesses the personality through the analysis of five factors of personality: neuroticism, extraversion, openness, kindness, and responsibility. Composed of 60 items (condensed version), this scale is answered by a five-point Likert scale, where 0 = strongly disagree and 4 = strongly agree. An example item is as follows: “Sometimes, when I read poetry or contemplate a work of art, I feel a deep emotion or excitement” [26].

Students' perceived academic performance represents the individual self-perception of the grade obtained in the simulation.

### 2.4. Data Analysis

The statistical analysis was carried out using the SPSS 23.0 statistical program. Descriptions were analyzed for each variable (*M* ± SD). First, the normality of the sample was tested by the Kolmogorov-Smirnov test. All the data presented a normal distribution. Then, to analyze the autonomic stress response during the hospital clinical simulation, a multivariate analysis of variance with the hospital clinical simulation moments as a fixed factor was conducted. The Tukey test was used for ad hoc comparisons. To analyze differences in the autonomic stress response during a hospital clinical simulation by the psychological profile, students were divided into groups by the percentile 50 in the psychological variables of SUDS, LET, PSS, and neuroticism factor, and differences between the high and low scores were tested by a *t*-test for independent samples. The significance level was *p* ≤ 0.05.

## 3. Results

We found a significant decrease in PNN50 between M3 and M0 and SD2 between M4 and M2. We did not find significant modifications in the other HRV parameters analyzed during the hospital clinical simulation (Table 2). The SUDS increased their value from 45.96 ± 22.10 to 59.41 ± 26.50 after the hospital clinical simulation.

The students analyzed were divided into two groups by the percentile 50 in SUDS, LET, PSS, and neuroticism to analyze differences between the high- and low-score groups. We found how high SUDS scores presented higher RMSSD and SD1 than lower scores; higher LET scores presented higher RMSSD, PNN50, SD1, SD2, and LF than lower LET scores; higher PSS scores presented higher RMSSD, PNN50, and SD1 than lower PSS scores; and higher neuroticism scores presented higher RMSSD than lower neuroticism scores (Table 3).

## 4. Discussion

The objectives of the present study were as follows: (1) to analyze the psychophysiological stress response of nurse degree students during a hospital clinical simulation and (ii) to analyze the effect of the psychological profile in the psychophysiological stress response of nurse degree students during a hospital clinical simulation. The initial hypothesis (i) was not fulfilled since no habituation was observed, and the second hypothesis (ii) was fulfilled since autonomic modulation was modulated by the LET, PSS, and neuroticism scores.

Analyzing the habituation process at the beginning of the simulation scenario (M0, M1), we observed low values in PNN50, RMSSD, and SD1 and high values in LF, data related with a high sympathetic activation [27]. This anticipatory anxiety response has been studied in recent years in high-demand environments such as sports, military, and academe [28], showing that coping with scenarios or events that are not under the control of the person directly impacts the autonomous response and may limit important capacities for the correct development of professional and academic activities. Specifically, interneuronal communication could be affected, negatively affecting executive functions such as reflection, abstraction, decision-making, or working memory, which are essential during the learning process [29]. Previous studies showed how the anticipatory anxiety response was modulated by experience, showing experienced participants' lower anticipatory anxiety response [11]. The no reduction in the anxiogenic response could be explained because the students were from the second course of the degree; then, they have no experience, and the expected habituation response was not reached.

Analyzing the habituation process during the last part of the clinical simulation (M2/M3/M4), we found a decrease in PNN50 (M3) and SD2 (M2/M3/M4) which indicates how students maintained their anxiety response, even increasing it at the end of the clinical practice. It was in line with the subjective perception of the distress of students since they indicate higher levels of SUDS at the end of the clinical practice than before starting. This difficulty in reducing the level of stress and alertness may be due to various factors, including certain personality traits that could impact this response [30].

Then, analyzing the psychological profile, we found a significantly high score in the N factor of NEO-FFI. These results are associated with emotional lability and difficulties in impulse control and restoring the psychological balance, being related to psychopathologies of different psychosomatic severities [31]. In this line, the score obtained highlighted that the coping strategies chosen have not been useful to achieve a reduction in the anxiety response. It is known that people who have a high rate tend to use styles of avoidance such as resignation, emotional discharge, or cognitive avoidance instead of styles based on analysis, positive evaluation, or the search for guidance [32], possibly due to the high perception of insecurity that these people present at the time of facing an event stressor as it is an evaluation.

In this line, a low LET score was observed. This scale provides a score on factors that are valuable for a person, indicating the average obtained by the group where they presented deficiencies in the participation of the person in tasks that determine the commitment to the lifetime [33, 34]. These data could be associated with the students' perception of uncontrollability, since when there are problems in recognition of valuable purposes, exposure to novel situations could make the subject consider the difficulties of the demands greater than their own resources to face [35–38]. In addition, we found how students with higher neuroticism, PSS, and LET scores presented a higher parasympathetic modulation. It seems that this psychological profile presented a higher autonomous response, showing psychological parameters to work with these students.

### 4.1. Practical Applications

The evaluation of the autonomic stress response could be analyzed to improve student stress management, improving their learning process and clinical skills. Students from other Health Science areas could use the present information to improve the preparation of clinical scenarios to control the psychophysiological stress response.

### 4.2. Limitation of the Study and Future Research Lines

The main limitation of this study was the small sample size due to the difficulty of recruiting students. In addition, the impossibility of measuring stress hormones (cortisol, adrenaline, alpha-amylase, etc.) to analyze the adrenergic stress response of students was also one of the main limitations. In addition, the sample has a wide variation in age and female gender predominance. As future research lines, we would propose to analyze the autonomic modulation in other degrees, as well as in other educational levels such as master and Ph.D. studies. In this line, correlation or regression analyses would also improve the relations between the psychological profile and the autonomic stress response.

## 5. Conclusion

A hospital clinical simulation produced a large sympathetic modulation of nurse students that was maintained during the entire clinical simulation. The autonomic response was modulated by the psychological profile of students, showing higher purpose in life, perceived stress, and neuroticism, presenting higher parasympathetic modulation.

## Figures and Tables

**Table 1:** Research design.

Psychological scales	SUDS	Hospital clinical simulation					SUDS
LET							
CFS		5 min pre	1/10	5/10	10/10	5 min post	Qualification student
PSS		M0	M1	M2	M3	M4	
Framingham Scale							
NEO-FFI		HRV					

M0 (5 min presimulation moment); M1 (interval corresponding to the first tenth of the hospital clinical simulation); M2 (interval corresponding to the fifth tenth of the hospital clinical simulation); M3 (interval corresponding to the tenth tenth of the hospital clinical simulation); and M4 5 (5 min postsimulation moment). SUDS: subjective scale of distress. Psychological scales: LET: Life Engagement Test; CFS: Coping Flexibility Scale; PSS: Perceived Stress Scale; HRV: heart rate variability.

**Table 2:** Differences in the measures through hospital clinical simulation.

	Prepractice	1/10	5/10	10/10	Postpractice	Chi^2^	Sig.	Post hoc
	M0	M1	M2	M3	M4			
LF (n.u.)	72.51 ± 15.85	73.62 ± 20.08	68.66 ± 15.70	73.50 ± 14.58	69.42 ± 13.77	5.156	0.272	
HF (n.u.)	27.40 ± 15.53	26.30 ± 20.01	31.21 ± 15.62	26.37 ± 14.48	30.48 ± 13.74	5.156	0.272	
LF/HF ratio (n.u.)	5.67 ± 8.53	5.06 ± 3.99	3.04 ± 2.00	4.87 ± 4.92	3.48 ± 3.66	5.156	0.272	
PNN50 (N°)	17.61 ± 13.74	16.22 ± 19.08	13.09 ± 17.47	13.83 ± 16.86	15.06 ± 15.19	14.180	0.015	3 < 0
RMSSD (ms)	44.45 ± 19.30	42.03 ± 36.96	39.88 ± 30.55	47.38 ± 40.90	39.12 ± 23.83	4.664	0.458	
SD1 (ms)	31.46 ± 13.66	29.86 ± 26.22	28.30 ± 21.69	28.04 ± 24.74	27.70 ± 16.86	7.778	0.100	
SD2 (ms)	113.73 ± 46.47	97.19 ± 43.21	82.72 ± 40.67	92.64 ± 41.88	116.35 ± 49.48	14.489	0.006	2 < 0; 3 < 0; 4 > 2

M: moment; HRV: heart rate variability; LF: the low-frequency band in normalized units (low-frequency); HF: the high-frequency band in normalized units (high-frequency); LF/HF ratio: a ratio of low frequency to high frequency; PNN50: the proportion of NN50 divided by the total number of NNs; RMSSD: square root of the mean of the sum of the squared differences between adjacent normal R-R intervals; n.u.: normalized unit; SD1: standard deviations of the scattergram 1; SD2: standard deviations of the scattergram 2.

**Table 3:** Differences between the low- and high-score groups in the variables analyzed.

		RMSSD	*p*	PNN50	*p*	SD1	*p*	SD2	*p*	LF	*p*	SUDS	*p*
Pre-SUDS	Low score	25.8 ± 16.0	0.015			25.8 ± 16.0	0.015						
	High score	64.1 ± 4.9				64.1 ± 4.9							
Post-SUDS	Low score											33.2 ± 17.3	0.002
	High score											77.5 ± 10.9	
LET	Low score	22.05 ± 3.24	0.023	22.05 ± 3.24	0.010	22.05 ± 3.24	0.025	22.05 ± 3.24	0.046	220.05 ± 30.24	0.035		
	High score	280.53 ± 10.17		280.53 ± 10.17		280.53 ± 10.17		280.53 ± 10.17		280.53 ± 10.17			
PSS	Low score	250.88 ± 20.97	0.026	250.88 ± 20.97	0.043	250.88 ± 20.97	0.006						
	High score	440.2 ± 70.04		440.2 ± 70.04		440.2 ± 70.04							
Neuroticism	Low score	130.94 ± 30.03	0.027										
	High score	300.92 ± 30.24											

LET: Life Engagement Test; PSS: Perceived Stress Scale; pre-SUDS: subjective measure of stress minutes before the simulation; post-SUDS: subjective measure of stress minutes after the simulation; LF: the low-frequency band in normalized units (low-frequency); PNN50: the number of successive differences of intervals which differ by more than 50 ms expressed as a percentage of the total; RMSSD: the square root of the average of the sum of the squared differences between adjacent normal R-R intervals; SD1: sensitivity of the short-term variability of the nonlinear spectrum of the HRV; SD2: sensitivity of the long-term variability of the nonlinear spectrum of the HRV.

## Data Availability

All data are in the manuscript.
